# Evaluating gaze behaviors as pre-touch reactions for virtual agents

**DOI:** 10.3389/fpsyg.2023.1129677

**Published:** 2023-03-06

**Authors:** Dario Alfonso Cuello Mejía, Hidenobu Sumioka, Hiroshi Ishiguro, Masahiro Shiomi

**Affiliations:** ^1^Interaction Science Laboratories, ATR, Kyoto, Japan; ^2^Intelligent Robotics Laboratory, Department of Systems Innovation, Graduate School of Engineer Science, Osaka University, Suita, Osaka, Japan; ^3^Hiroshi Ishiguro Laboratories, ATR, Kyoto, Japan

**Keywords:** virtual reality, gaze behavior, pre-touch behavior, computer-human interaction, virtual human

## Abstract

**Background:**

Reaction behaviors by human-looking agents to nonverbal communication cues significantly affect how they are perceived as well as how they directly affect interactions. Some studies have evaluated such reactions toward several interactions, although few approached before-touch situations and how the agent’s reaction is perceived. Specifically, it has not been considered how pre-touch reactions impact the interaction, the influence of gaze behavior in a before-touch situation context and how it can condition the participant’s perception and preferences in the interaction. The present study investigated the factors that define pre-touch reactions in a humanoid avatar in a virtual reality environment and how they influence people’s perceptions of the avatars.

**Methods:**

We performed two experiments to assess the differences between approaches from inside and outside the field of view (FoV) and implemented four different gaze behaviors: face-looking, hand-looking, face-then-hand looking and hand-then-face looking behaviors. We also evaluated the participants’ preferences based on the perceived human-likeness, naturalness, and likeability. In Experiment 1, we evaluated the number of steps in gaze behavior, the order of the gaze-steps and the gender; Experiment 2 evaluated the number and order of the gaze-steps.

**Results:**

A two-step gaze behavior was perceived as more human and more natural from both inside and outside the field of view and that a face-first looking behavior when defining only a one-step gaze movement was preferable to hand-first looking behavior from inside the field of view. Regarding the location from where the approach was performed, our results show that a relatively complex gaze movement, including a face-looking behavior, is fundamental for improving the perceptions of agents in before-touch situations.

**Discussion:**

The inclusion of gaze behavior as part of a possible touch interaction is helpful for developing more responsive avatars and gives another communication channel for increasing the immersion and enhance the experience in Virtual Reality environments, extending the frontiers of haptic interaction and complementing the already studied nonverbal communication cues.

## 1. Introduction

Designing natural reaction behaviors is critical to develop human-like virtual agents. In human interaction, nonverbal communication cues offer important information about a person’s purpose and provide a way to express feelings about it. For example, during an interaction, the way that someone is looking at her conversation partner is used by that partner to collect information, regulate the interpersonal status, and prepare a reaction (Abele, 1986). The surrounding space is managed as a reaction that reflects the interpersonal relationship and the degree of intimacy (Hall et al., 1968). Touch interactions, in both proactive and reactive behaviors, affect mental and physical well-being (Field, 2001). Therefore, providing human-like avatars in virtual scenarios with such abilities can drastically improve interaction experiences.

Past studies considered and developed several reactions toward multimodal actions with users for improving agents’ behaviors. For example, the gaze behaviors of agents can change their impressions in human-robot interaction (Hirano et al., 2018) and the same can be used in virtual agents for emotional expressions (Lance and Marsella, 2008) and to define the conversational flow (Pejsa et al., 2017). The interaction distance is another factor that conditions behaviors. Some similarities with the real world have been found, where people managed the same interaction distance in an augmented reality environment, recognizing an agent’s personal space (Huang et al., 2022), and showing a similar handling of space as if approaching a human (Bailenson et al., 2003). Considering these similarities, virtual reality is being used for evaluating how the emotional states of agents affect personal space (Bönsch et al., 2018). Incorporating touch interactions into the design of agents might provide a powerful tool for conveying emotions. For example, the inclusion of haptic interaction improved nonverbal communication in a doctor-patient simulated situation (Kotranza et al., 2009).

However, one essential but missing reaction behavior is a pre-touch reaction behavior. Although past studies in human-agent interaction reported the importance of before-touch reactions, they focused on the reaction distance rather than the reaction behavior. For example, some studies defined a pre-touch reaction distance for the face in both the real world with a human-like robot (Shiomi et al., 2018) and in a virtual reality environment with a human-like agent (Mejía et al., 2021). Another study analyzed human interaction and defined pre-touch distances for socially touchable upper body parts (Cuello Mejía et al., 2021). These studies defined the distances for each socially-accepted body part for touch interactions without considering how the agent should react *before* a touch interaction. This response can be useful for extending the capabilities of touch interaction, giving a proper context, and providing enough information for adapting to the situation. With the purpose of improving human-agent interaction, implementing a human-like acceptable behavior is essential. Thus, searching for more information within the non-verbal communication cues and evaluating and developing how an agent should react when a touch interaction is attempted before the act of touching is performed could give hints about how the interaction should evolve and how the agent should react towards better communication.

Reaction and nonverbal communication cues are interrelated and significantly influenced by the information available in the environment. This means that gaze behavior should be part of the expected reactions from avatars. In pre-touch situations, the available visual information is essential for starting and developing interactions, and the FoV plays an important role, defining the area within which such data can be acquired. This region is fundamental because it determines the reaction to the same interaction attempt, depending on whether it comes from inside or outside the FoV. Implementations have identified an FoV effect for the deployment of therapy systems (Anders et al., 2007) and the spatial processing of near and far spaces using virtual reality systems (Beck et al., 2007). FoV also directly affects the experience in a virtual reality environment and the feelings of presence and enjoyment (Lin et al., 2002).

**Figure 1:**
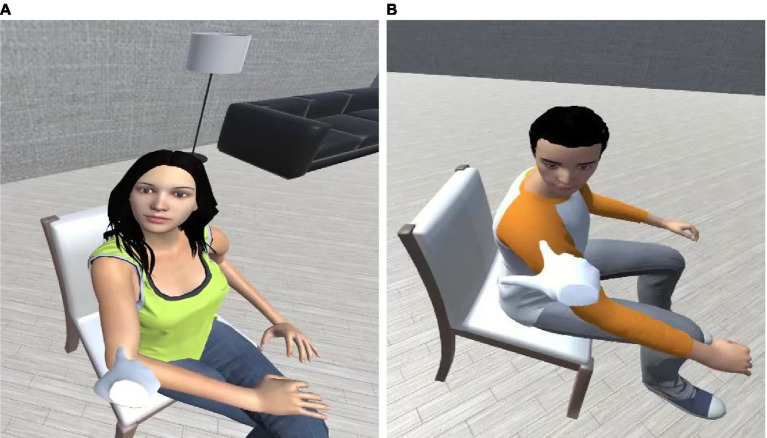
Two looking behaviors: **(A)** a female avatar’s pre-touch reaction face and **(B)** a male avatar’s pre-touch reaction hand.

This study focuses on designing reaction behaviors for pre-touch situations and analyzes how such behaviors affect impressions of avatars. As a first step for achieving natural reaction behaviors, we focused on gaze behavior design, proxemics space awareness and pre-touch context, and considered the FoV as an influencing factor. We conducted two experiments: the first evaluated different gaze behaviors and an agent’s gender effect (Figure 1) when the touch interaction is attempted from inside the agent’s FoV. Experiment 2, based on the results of Experiment 1, evaluated the differences in gaze behavior when the touch attempt is initiated from outside the agent’s FoV.

## 2. Theoretical background

### 2.1. Awareness, gender, and gaze behavior effect in human interaction

According to the intimacy equilibrium model, nonverbal communication cues such as proxemics and gaze behavior maintain a balance in their expressions (Argyle and Dean, 1965). For example, if the interpersonal distance is low, eye-contact behavior will decrease. This applies to any kind of interaction in society. However, considering the development of new technologies that allow people to interact in different atypical contexts, such as virtual reality (VR) scenarios, how these social interaction rules apply to these new environments must be studied and evaluated.

How people manage their surrounding space plays an important role in any interaction. Proxemics is the study of perception and the use of space that depends deeply on what kind of interaction we are experiencing and with whom it is going to be performed, defining different spaces for different social situations (Hall et al., 1968). This means that body posture, alignment, the distance itself, and so on are involved in any human interaction and provide the means for predicting, adapting, and preparing a proper reaction. The concept of proxemics provides several interaction distances that vary depending on the situation and the relationships (Hall et al., 1968). These distances, which are applied in both verbal and nonverbal communication, must be analyzed to reveal how they affect interactions. For example, proxemics and touch interaction, which are closely related, are used for expressing power and control in both positive and negative scenarios (Andersen et al., 2013). The combination of proxemics and gaze behavior also provides a way to compensate for the interaction. Interpersonal gaze is reduced as a response to a closer interaction distance (Rosenfeld et al., 1984).

Although proxemics defines a range of distances for social interaction, all depend on several cultural variables. For example, some studies have found that gender plays an important role. With strangers, interpersonal distance during verbal communication is greater for women than men (Heshka and Nelson, 1972). A more recent study identified a relationship between arm length and gender, concluding that the effect of gender in proxemics can also have a biological explanation based on body structure (Bruno and Muzzolini, 2013). Other studies involving human-looking agents offer diverse results. In some, gender had no effect on the preference of the reaction distance in an attempted touch interaction with a female-looking android (Shiomi et al., 2018) or in a VR environment (Mejía et al., 2021); in another study, the interpersonal distance was affected by the avatar’s gender in a VR environment (Bailenson et al., 2003).

On the other hand, looking behavior is one of the most commonly used cues for conveying intentions without involving too much effort or movement. In human relationships, the gaze direction indicates the interaction’s main focus (Mareschal et al., 2013) and can provide information on how people interact in society and identify differences between individuals who might struggle during social interactions (Gamer et al., 2011). This behavior is not exclusive to humans, although it is much more complex than in other mammals or animals and involves more than the eyes (Emery, 2000). Therefore, gaze behavior provides information for the recognition of an approach intention and fuels a reaction before any other interaction is carried out, such as a touch. Gaze direction offers a tool with which an interaction’s attention can be controlled. In a conversational context, it can bring attention to something outside the participants (Richardson et al., 2009; Cummins, 2012) and be used for taking turns while being noticed by the other part (Waters et al., 1998; Hessels, 2020). Looking behavior is also used for communicating intention, monitoring the reactions of other participants, and regulating interactions (Abele, 1986). This means that gaze behavior during an interaction can be used for learning when and how to continue or finish an interaction by involving direct eye contact or looking at a body part or an object to distract the focus away and onto something else.

Previous research studied interpersonal distances in a virtual reality context for obtaining appropriate reaction distances for a touch attempt (Mejía et al., 2021) and compared them with real life ones (Bailenson et al., 2003). Other studies evaluated the cultural differences in the usage of personal space by VR (Hasler and Friedman, 2012). Unfortunately, no previous work has addressed how an avatar’s nonverbal communication behavior can condition perceptions. Looking behavior has also been studied from a cultural perspective using VR (Haensel et al., 2022) and how it can be used to influence a participant to accept or decline an economical giveaway (Harjunen et al., 2018). Also, agents embodiment and perceived extroversion of virtual agents are affected by gaze behavior (Koda and Ishioh, 2018), and as for the human side, tracking human eye gaze has improved the immersion and interaction with a virtual agent in a VR environment (Kevin et al., 2018). But again, these studies did not evaluate the avatar’s behavior as a reaction to a communicative attempt from a participant. Few studies have defined avatar behaviors for before-touch interactions. Some research defined a pre-touch reaction distance that determines which body parts are socially touchable in the physical world (Cuello Mejía et al., 2021). Yet the same problem remains: how should an avatar behave when it attempts a touch interaction? What factors determine preferences? How do such preferences affect impressions toward it? Considering a human-like avatar, the reaction expectancy might be conditioned on how humans interact. In human interaction, looking behavior is a complex task that involves face allocation (Hessels et al., 2017) and depends on the task being performed (Hessels et al., 2019). Sometimes gender plays an important role in defining interpersonal distances (Heshka and Nelson, 1972), and looking behavior can be used to focus attention on someone or something else (Böckler et al., 2015). In other words, the gaze is a complex behavior that is constantly changing and adapting, depending on the context, the type of interaction, and both the people and the objects that are involved. Therefore, for a human-like avatar, gaze behavior reaction to a touch attempt should include looking at the approaching hand and face-looking movements. Based on this context, we propose the following hypothesis:

*H1*: A complex two-step gaze behavior will be preferred over a simple, one-step gaze behavior.

### 2.2. Importance of field of view in human behavior

Visual information is fundamental for an adequate human interaction. The field of view (FoV) is defined as the area where human eyes can gather data given a moment of time, providing peripheral information and help to identify the shapes, positions, and structures of the objects within it. Peripheral vision provides the foundations for capable performance in such basic actions as walking, reaching, body posture, and interaction with others (Alfano and Michel, 1990). FoV plays a critical role in any kind of interaction that can be done, including pre-touch interactions.

Several studies have evaluated the effects of changes in FoV as well as its importance. For example, a reduction in the size of FoV caused by aging can be partially recovered (Ball et al., 1988). FoV’s restriction and reduction can lead to performance reduction and body discomfort, such as dizziness and disorientation (Alfano and Michel, 1990). Other studies found that reduced peripheral vision lowered spatial learning (Barhorst-Cates et al., 2016), reduced maneuvering speed and accuracy (Toet et al., 2007), and damaged the target location of hazard perception (Shahar et al., 2010).

The effect of FoV on concentration is also noticeable. Humans tend to focus their attention on the center of an image either for starting their visual exploration or for early processing of situations (Tatler, 2007). On the other hand, occluding the central area and leaving only peripheral information can lead to overestimation of motion speed (Pretto et al., 2009). This means that the data gathered through our cone of vision is fundamental for proper social interactions. Such information is actively used for the development of real world and virtual reality systems. For example, the central fixation bias has been used for developing telepresence systems with robots with narrower FoVs to improve interactions (Kiselev et al., 2014). In virtual reality environments, a higher FoV can enhance performance in visual scanning tasks (Ragan et al., 2015) as well as increase simulation sickness (Seay et al., 2002). All these researches describe the importance of FoV in surrounding space interactions and conclude that the effect of a stimuli is greatly related to peripheral vision and where the stimuli is coming from: inside or outside the FoV.

Eye contact, which is a fundamental part of human interaction, can focus attention to enhance the perception of the people involved (Senju and Johnson, 2009) and how the FoV condition affects the interaction of people with their surroundings. Therefore, it would be interesting to evaluate the effect of direct eye-looking behaviors from different perspectives: inside and outside the FoV. Since peripheral vision influences the sense of presence in VR environments (Lin et al., 2002), avatar reactions might differ based on FoV. Some studies have shown how peripheral stimuli attract reflexive and involuntary attention and how this result can guide attention in such scenarios as sports (Schumacher et al., 2019) and panoramic videos (Schmitz et al., 2020), Almost every time a stimuli or an object enters the FoV, it catches the attention. The expected reaction from the avatar must resemble the one performed by humans: looking directly at the new object inside the FoV. Therefore, we made the following hypotheses:

*H2*: The FoV affects the preferred gaze behavior.

*H2a*: Inside the avatar’s FoV, a “first face” looking behavior will be preferred.

*H2b*: Outside the avatar’s FoV, a “first hand” looking behavior will be preferred.

## 3. Materials and methods for Experiment 1

Experiment 1 is based on our first hypothesis. At the beginning of this study, we chose to evaluate different before-touch reactions and how they impacted our participant’s perceptions of the avatar.

### 3.1. Conditions

For evaluating the first hypothesis, we considered the number of *gaze-steps* as a pre-touch reaction (*one-step*/*two-step*), the *face-first* (*face-/hand-*first looking behavior), and the avatar’s *gender* (*male*/*female*). For the hand that made the attempt to touch, since most people are right-handed (Sato et al., 2008), our participants were told to use their right hands. Experiment 1 evaluated the following conditions:

#### 3.1.1. Gaze-step

We defined two looking behaviors per *gaze-step* condition. For the *one-step* conditions: (1a) look directly at the participant’s face and (1b) look directly at the participant’s right hand. For the *two-step* conditions: (2a) look at the participant’s face for 1 scond and then at the participant’s right hand and (2b) first look at the participant’s right hand for 1 s and then at the participant’s face. The duration of the looking behavior was based on different works involving reactions to visual stimulation and other VR environments. In the case of reaction to visual stimuli, reaction times of gaze behavior involving face position and gaze aversion were between 920 and 1,000 ms (Böckler et al., 2015) and, in the case of studies involving virtual avatars, gaze behaviors such as “look-away” and “gaze-at” had similar timing for evaluating the interaction with a virtual avatar (Koda et al., 2017).

#### 3.1.2. Face-first

We also analyzed the same looking conditions by considering the order of the looking behaviors. These are the *face*-looking conditions: (f1) look at the participant’s face and (f2) first look at the participant’s face and next at the participant’s right hand. (These are the same (1a) and (2a) conditions described in the *gaze-step* condition.) These are the *hand*-looking conditions: (h1) look at the participant’s right hand and (h2) first look at the participant’s right hand and next at the participant’s face. (These are the same (1b) and (2b) conditions described in the *gaze-step* condition.)

#### 3.1.3. Gender

Since some studies involving human-looking agents show diverse results regarding gender effects as well as differences in the results that compare human and human-agent interactions, we included the avatar’s gender as a condition: (1) a male avatar and (2) a female avatar.

### 3.2. System

We used Unity (Unity Technologies, n.d.) as the development platform for implementing our virtual environment and the avatars’ behaviors. We deployed our system with Oculus Rift S (“Oculus Rift S: PC-Powered VR Gaming Headset | Oculus”, n.d.) because it can easily connect with Unity and implement the desired behaviors. For the avatars, we used two 3D models from the Unity Assets Store (o3n Studio, n.d.), and implemented independent animations for the gaze behaviors, and used animations from Adobe Mixamo (“Mixamo”, n.d.) for the idle avatar movements (e.g., breathing movement) to add some natural feeling to their behavior.

For the avatar reaction, we implemented a face-looking reaction behavior at a certain distance from the participant’s hand, based on the previous work (Cuello Mejía et al., 2021). In a previous study, pre-touch reaction distances were obtained for socially-touchable upper body parts: shoulders (24.8 cm), elbows (24.1 cm), and hands (21.5 cm) based on human interactions. We also implemented an “awareness” behavior for the avatar based on the concept of proxemics (Hall et al., 1968) and defined a looking behavior for it when the participant enters its personal space (~1.2 m).

### 3.3. Procedure

**Figure 2:**
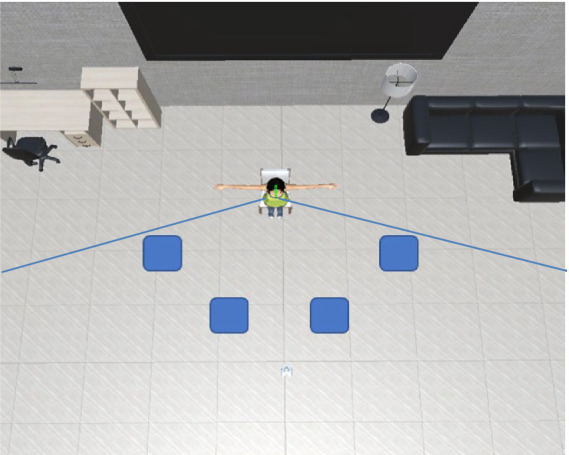
Virtual reality setup for first stage of experiment: avatar is facing participant located in four different places inside avatar’s FoV (blue boxes).

We designed the VR environment shown in Figure 2. The avatar is located in the center of a room, sitting on a chair and facing the participant. The participant was placed in four locations, all standing approximately 3.0 m away and inside the avatar’s FoV. We asked our participants to approach the avatar and slowly try to touch with their right hand one of three body parts of the avatar: a shoulder, an elbow, or a hand. As explained in the system (subsection 3.2), the avatar will react to the participant at a certain distance. We explained that this behavior should be understood as a stop signal and that there would be no touch interaction with the agent. Next, the participants pressed a button on the controller to reset their position and restart the approaching movement. The conditions are shown in Table 1: two avatar’s genders and four gaze behaviors. All of these were evaluated from four different locations.

**Table 1:** Conditions and locations for Experiment 1.

Gender		Gaze Behavior			
Male	Female	Face	Hand	Face-Hand	Hand-Face

### 3.4. Avatar behavior evaluation

**Figure 3:**
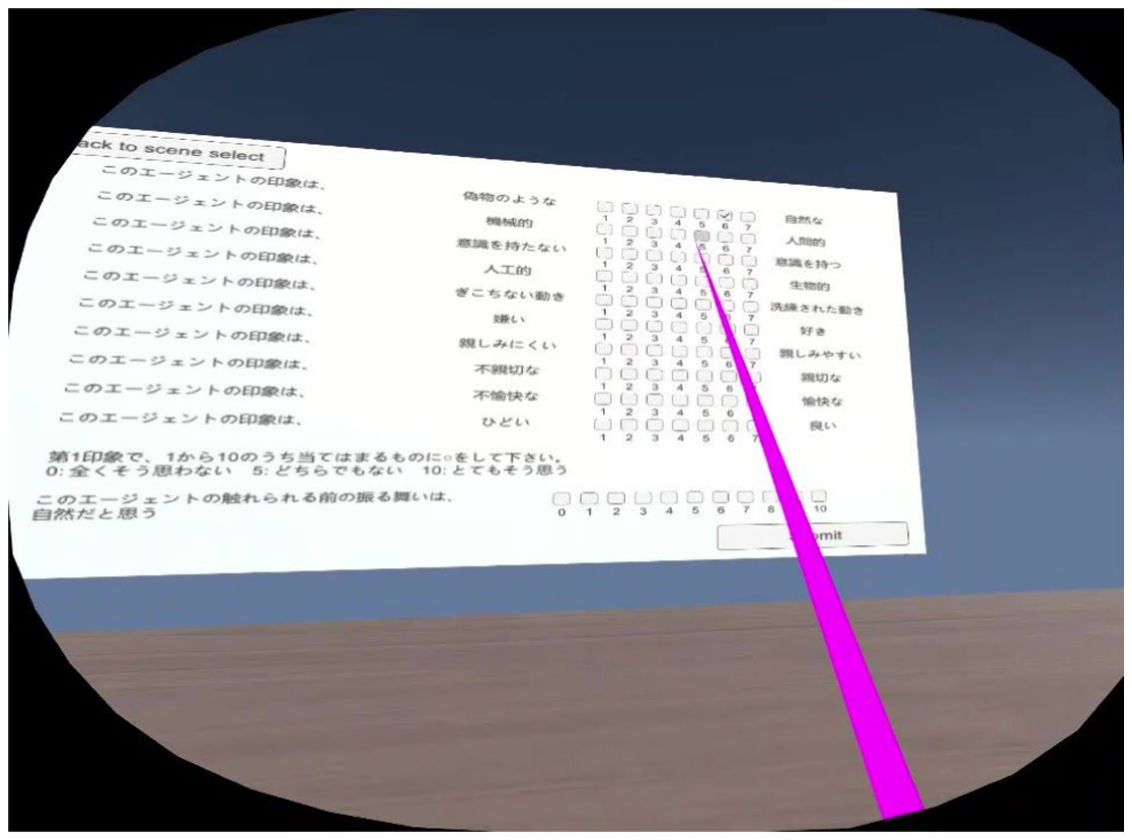
Completing a questionnaire in VR environment.

After evaluating each set of conditions from the four defined positions, the participants completed a questionnaire, which was implemented inside the VR system to make the experiment more immersive and comfortable for them and to save time. A screenshot of the questionnaire is shown in Figure 3.

Since we wanted to evaluate the participants’ preferences and perceptions, we used Godspeed questionnaires for anthropomorphism and likeability (Bartneck et al., 2009), and made an 11-point response format for naturalness evaluation (Wu and Leung, 2017). The questionnaire used for this experiment both in Japanese and English is included as Supplementary material. The Cronbach-alpha value for the Godspeed questionnaire used was calculated for both anthropomorphism (*α* = 0.924) and likeability (*α* = 0.914) items for validating the reliability of the results for our particular case (Cronbach, 1951). The participants used the Oculus Rift S controller to point and selecting their answers. Once all the questions were answered, the participants clicked the “Submit” button and finally the “Return to the scene” to continue the experimental procedure.

### 3.5. Participants

Experiment 1 included 29 Japanese participants: 15 males and 14 females with ages between 21 and 58 years old (mean = 40.46, SD = 12.28). We explained the experiment’s steps to them, asked them to put on the HMD, and showed them how to use its controllers. We monitored their states and allowed them to stop whenever they wanted. The ethics committee at the Advanced Telecommunication Research Institute (ATR) approved this paper’s methodology (21-501-4).

## 4. Results and discussions for Experiment 1

### 4.1. Questionnaire results

**Figure 4:**
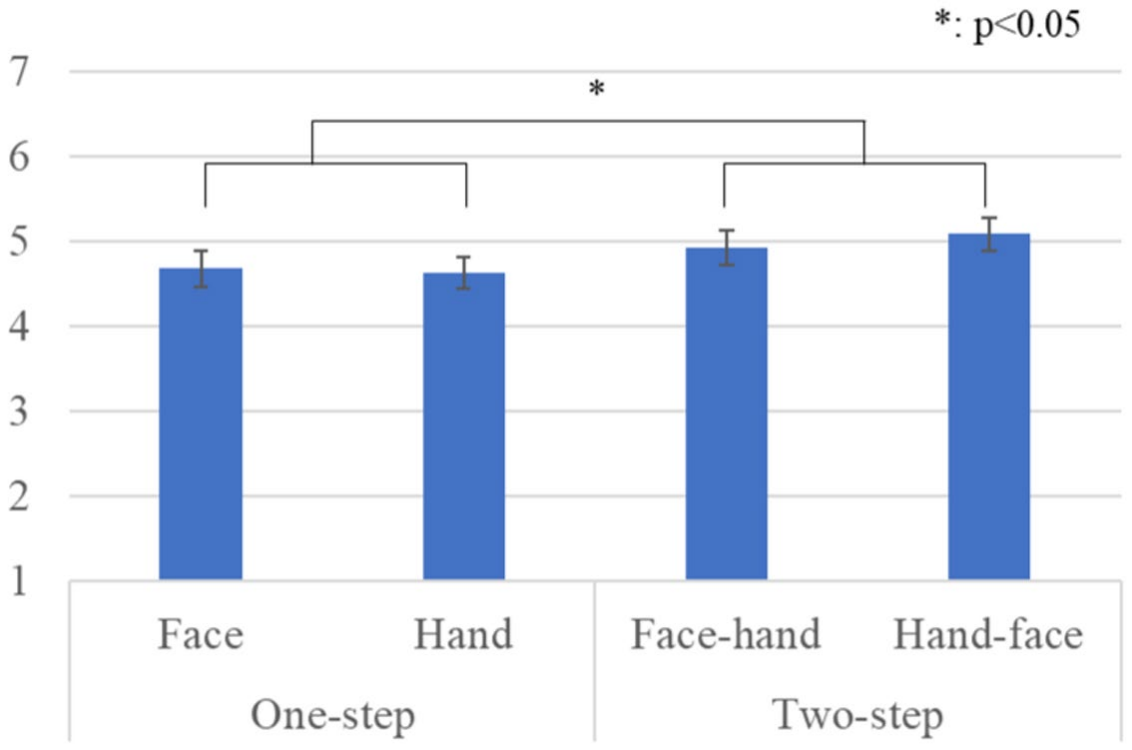
Questionnaire scores and S.E. for anthropomorphism: Since gender factor had no significant differences, we omitted it from this graph.

Figure 4 shows the questionnaire results and the standard error (S.E.) of the anthropomorphism scale. We conducted a repeated three-factor ANOVA whose results showed a significant difference in the *gaze-step* factor [*F*(1, 28) *=* 6.567, *p =* 0.016, *partial η^2^ =* 0.190]. We did not find any significant differences in the *face-first* factor [*F*(1, 28) *=* 0.299, *p =* 0.589, *partial η^2^ =* 0.011], in the *gender* factor [*F*(1, 28) *=* 2.289, *p =* 0.141, *partial η^2^ =* 0.076], in the simple interaction effect between the *gender* and *face-first* factors [*F*(1, 28) *=* 0.024, *p =* 0.877, *partial η^2^ =* 0.001], in the simple interaction effect between the *gender* and *gaze-step* factors [*F*(1, 28) *=* 1.682, *p =* 0.205, *partial η^2^ =* 0.057], in the simple interaction effect between *face-first* and *gaze-step* factors [*F*(1, 28) *=* 1.675, *p =* 0.206, *partial η^2^ =* 0.056], or in the two-way interaction effect [*F*(1, 28) *=* 0.242, *p =* 0.626, *partial η^2^ =* 0.009].

**Figure 5:**
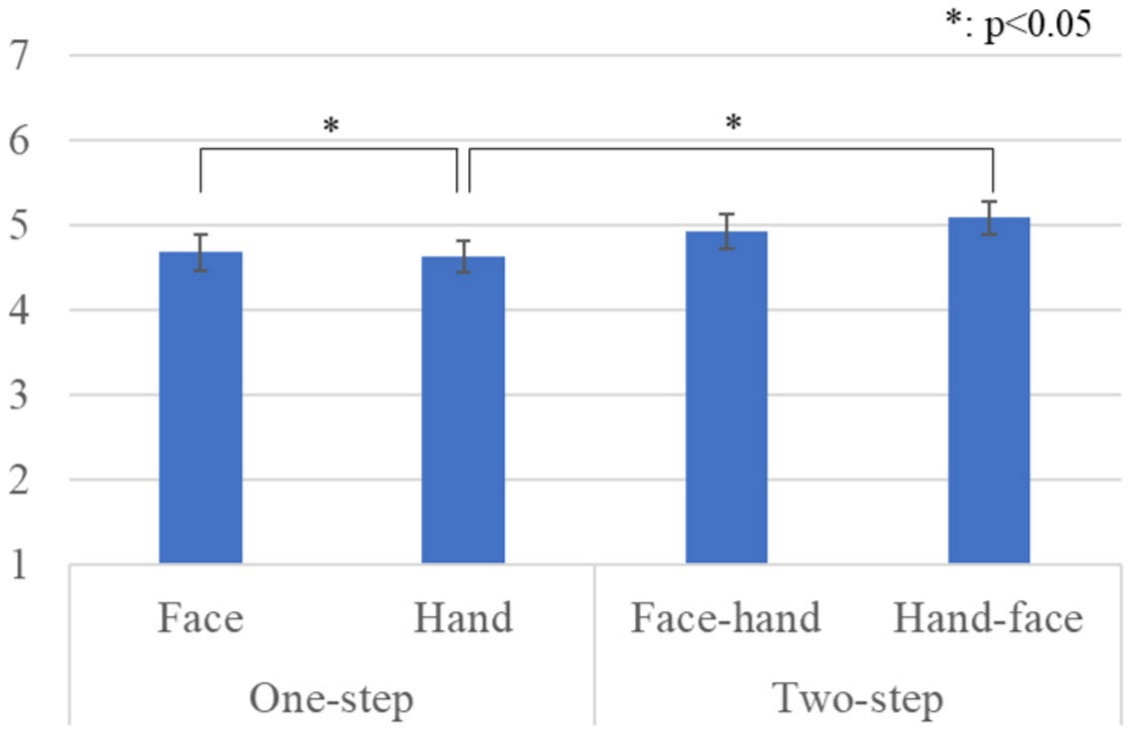
Questionnaire scores and S.E. for likeability: Since gender factor had no significant differences, we omitted it from this graph.

Figure 5 shows the questionnaire results and the standard error (S.E.) of the likeability scale. We conducted a repeated three-factor ANOVA whose results showed significant differences in the *gaze-step* factor [*F*(1, 28) *=* 5.050, *p =* 0.033, *partial η^2^ =* 0.153] and a simple interaction effect between the *face-first* and *gaze-step* factors [*F*(1, 28) *=* 6.478, *p =* 0.015, *partial η^2^ =* 0.194]. We did not find any significant differences in the *face-first* factor [*F*(1, 28) *=* 0.846, *p =* 0.365, *partial η^2^ =* 0.029], in the *gender* factor [*F*(1, 28) *=* 0.041, *p =* 0.841, *partial η^2^ =* 0.001], in the simple interaction effect between the *gender* and *face-first* factors [*F*(1, 28) *=* 1.937, *p =* 0.175, *partial η^2^ =* 0.065], in the simple interaction effect between the *gender* and *gaze-step* factors [*F*(1, 28) *=* 0.119, *p =* 0.732, *partial η^2^ =* 0.004], or in the two-way interaction effect [*F*(1, 28) *=* 0.919, *p =* 0.346, *partial η^2^ =* 0.032]. The simple main effects showed a significant difference: *two-step* > *one-step, p =* 0.012 in the *hand* condition. The simple main effects also showed a significant difference: *face* > *hand, p =* 0.049 in the *one-step* condition.

**Figure 6:**
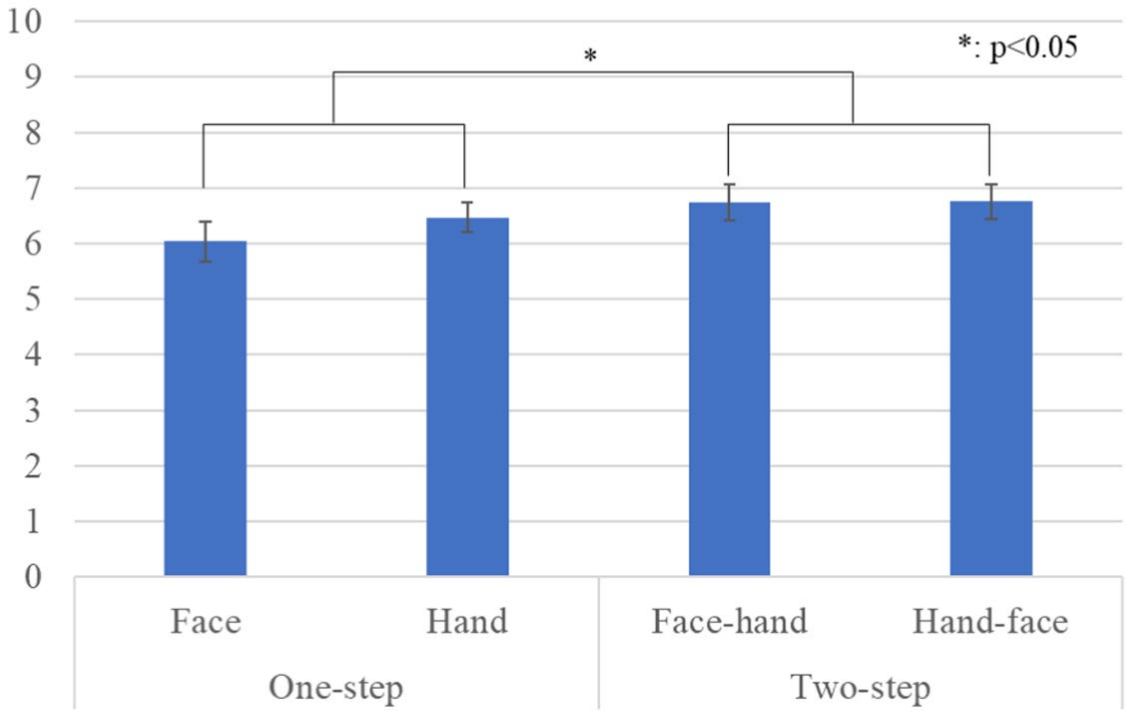
Questionnaire scores and S.E. for naturalness: Since gender factor had no significant differences, we omitted it from this graph.

Figure 6 shows the questionnaire results and the standard error (S.E.) of the naturalness scores. We conducted a repeated three-factor ANOVA whose results showed a significant difference in the *gaze-step* factor [*F*(1, 28) *=* 5.273, *p =* 0.029, *partial η^2^ =* 0.158]. We did not find any significant differences in the *face-first* factor [*F*(1, 28) *=* 1.442, *p =* 0.240, *partial η^2^ =* 0.049], in the *gender* factor [*F*(1, 28) *=* 0.415, *p =* 0.525, *partial η^2^ =* 0.015], in the simple interaction effect between the *gender* and *face-first* factors [*F*(1, 28) *=* 1.068, *p =* 0.310, *partial η^2^ =* 0.037], in the simple interaction effect between the *gender* and *gaze-step* factors [*F*(1, 28) *=* 3.936, *p =* 0.057, *partial η^2^ =* 0.123], in the simple interaction effect between the *face-first* and *gaze-step* factors [*F*(1, 28) *=* 1.433, *p =* 0.241, *partial η^2^ =* 0.049], or in the two-way interaction effect [*F*(1, 28) *=* 0.197, *p =* 0.661, *partial η^2^ =* 0.007].

### 4.2. Discussion

Our results showed that a two-step looking behavior was perceived as more human-like, more natural, and more likable than just a one-step looking behavior. For the likeability score, the *face-first*-looking behavior was particularly important. It also affected the preferred *gaze-step*; *two-steps* for gaze behavior were preferred in the hand condition, and the face-looking behavior was preferred over the hand-looking behavior in the *one-step* gaze behavior condition. In social interactions, gaze serves multiple uses: monitoring the interlocutor behavior, communicating one’s status, and checking probable new interactions (Abele, 1986). All these behaviors need continuous eye movement in combination with other cues, such as facial expressions and body movements, which is a fairly complex combination. Having an overly simple behavior might cause a participant to feel that she is not interacting with a human-like entity. Expecting a more complex and elaborate reaction based on avatar’s human-like appearance could have influenced their preferences. Furthermore, we found no significant difference in the *gaze-step* order. This means that the participants did not believe that it was important whether the avatar made direct eye contact at the beginning or the end of the interaction. However, considering that the two-step gaze behavior was preferred and that all the cases that involved face-looking behavior were also preferred, we assume that eye contact is fundamental for a proper interaction with a human-like avatar.

Gender did not affect the perception of the avatar’s human-likeness and naturalness; it made it neither more nor less likeable. Although some studies identified a difference in how the same and different genders manage personal space (Heshka and Nelson, 1972) and how they interact with human-looking agents as a before-touch reaction around the face (Shiomi et al., 2018), other studies concluded that gender made no difference in such preferences. For example, when analyzing the minimum comfortable distance for socially-touchable body parts in human interaction for implementation in a humanoid robot, gender did not affect the obtained values (Cuello Mejía et al., 2021). Also, in a virtual reality environment, this minimum distance around the face was not strongly affected by the gender of the avatar or the participant (Mejía et al., 2021).

## 5. Materials and methods for Experiment 2

In human interaction, the reaction from a touch attempt can drastically change if it is performed outside our sight (Hall et al., 1968). After the first experiment and analyzing its data, we wanted to identify whether the FoV significantly influenced the participant’s preferences, leading to our second hypothesis. Therefore, Experiment 2 evaluated whether this case is also applicable to humanoid avatars in virtual reality environments.

### 5.1. Conditions

Based on the findings of previous related studies (Cuello Mejía et al., 2021; Mejía et al., 2021), where gender did not significantly affect the results and also considering our results in Experiment 1, we removed the gender factor. Therefore, for Experiment 2, we used an avatar of the same gender as the participant. For the gaze behavior, since eye contact is fundamental in human interaction (Emery, 2000), we wanted to evaluate whether this idea also applies when the touch interaction is attempted from outside the avatar’s sight. As in Experiment 1, the participants used their right hand for the touch approach. We evaluated the following conditions:

#### 5.1.1. Gaze-step

We defined two looking behaviors per *gaze-step* condition. For the *one-step* conditions: (1a) look directly at the participant’s face and (1b) look directly at the participant’s right hand. For the *two-step* conditions: (2a) look at the participant’s face for 1 s and look at the participant’s right hand and (2b) first look at the participant’s right hand for 1 s and then look at the participant’s face.

#### 5.1.2. Hand-first

We analyzed the same looking conditions considering the order of the looking behavior. The *hand*-looking conditions: (h1) look at the participant’s right hand, and (h2) first look at the participant’s right hand and then the participant’s face. (These are the same (1b) and (2b) conditions described in the *gaze-step* condition.) The *face*-looking conditions: (f1) look at the participant’s face and (f2) first look at the participant’s face and the participant’s right hand. (These are the same (1a) and (2a) conditions described in the *gaze-step* condition.)

### 5.2. System

For Experiment 2, we used the same system: Unity as the development platform, Oculus Rift S as the implementation hardware, and the same male and female 3D models from the Unity Assets Store with Adobe Mixamo sitting animations to improve their immersion into the environment. As for the avatar reaction behaviors, we used the same reaction distances for the body parts: shoulder (24.8 cm), elbow (24.1 cm), and hand (21.5 cm). We also removed the “awareness” behavior because this action is not natural in an interaction where the avatar cannot see the approach.

### 5.3. Procedure

**Figure 7:**
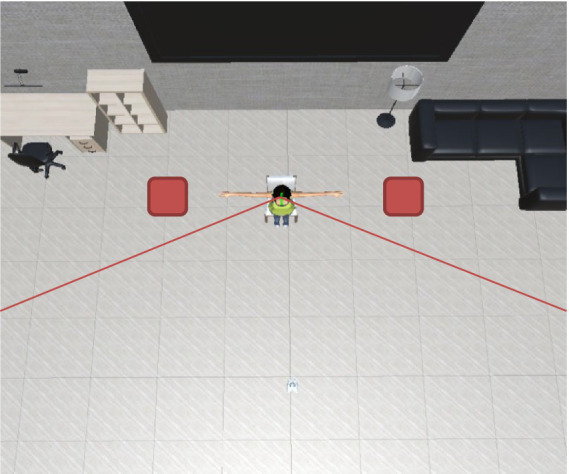
Virtual reality setup for the second stage of the experiment: the avatar is facing the center of the room, and the participant is located outside the avatar’s FoV (red boxes).

For this experiment, we modified the first VR setup shown in Figure 7. The avatar is sitting in the center of the room, as in the previous setup. The difference is that in this case, the approach came from the avatar’s sides, and so it is not “aware” of the participant’s approach. Since the participant was standing 1.0 m from the avatar, no extra movement was needed. As in Experiment 1, the participants slowly extended their right hand to attempt touching one of the three body parts mentioned above at the same reaction distances for each body part that were previously used and stating that there would be no touch interaction with the agent. The conditions evaluated were four gaze behaviors from two different locations (Table 2).

**Table 2:** Conditions for Experiment 2.

Gaze Behavior			
Face	Hand	Face-Hand	Hand-Face

### 5.4. Avatar evaluation behavior

As in Experiment 1, after every condition, we evaluated the participants’ preferences and perceptions using the Godspeed questionnaire for anthropomorphism and likeability (Bartneck et al., 2009) and an 11-point response format for naturalness rating (Wu and Leung, 2017). We used the same questionnaire, and the same steps were performed. The Cronbach-alpha value was also calculated for both anthropomorphism (*α* = 0.861) and likeability (*α* = 0.845) items. After the participants answered all the questions, they pressed a button to continue to the next condition.

### 5.5. Participants

For Experiment 2, 42 Japanese participants joined: 21 males and 21 females with ages between 20 and 59 years old (mean = 37.90, SD = 11.80). As in Experiment 1, we explained the procedure to them, asked them to put the HMD on, and showed them how to use the controllers. Although the procedure was shorter than the first experiment, we continuously checked the comfort of the participants and reminded them that they could stop the experiment at any time. Experiment 2’s participants were different from those in Experiment 1. The ethics committee at the Advanced Telecommunication Research Institute (ATR) approved this paper’s methodology (21-501-4).

## 6. Results and discussion for Experiment 2

### 6.1. Questionnaire results

**Figure 8:**
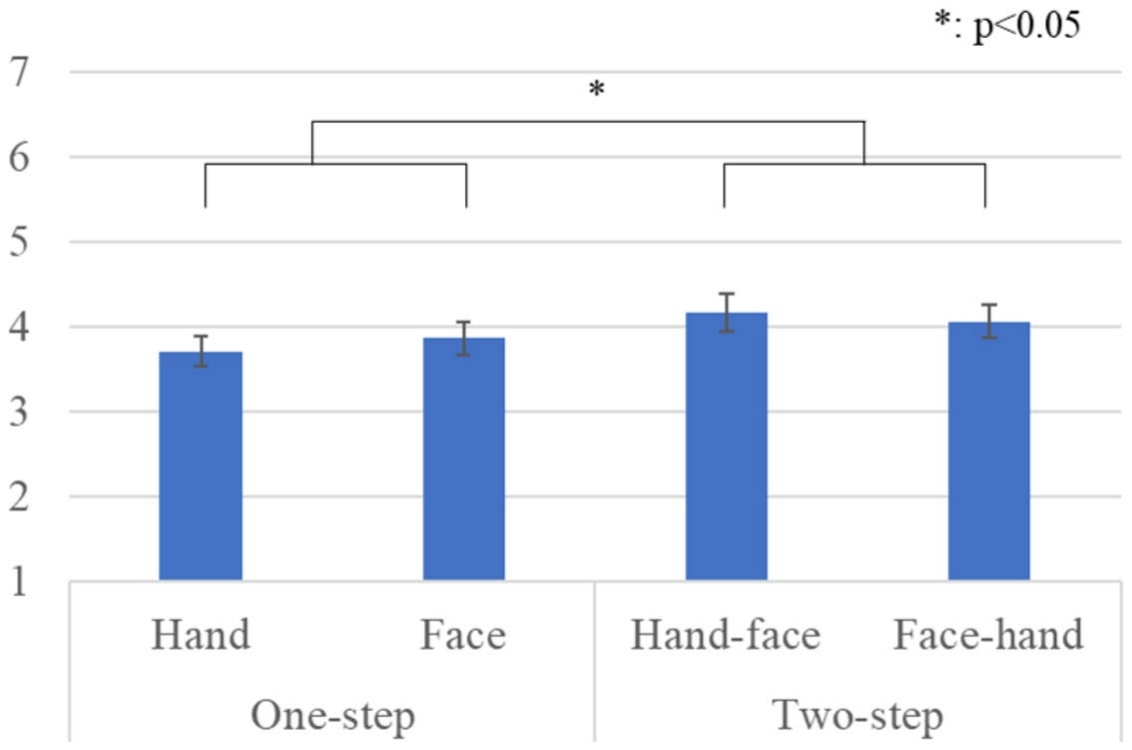
Questionnaire scores and S.E. for anthropomorphism.

Figure 8 shows the questionnaire results and the standard error (S.E.) of the anthropomorphism scale. We conducted a repeated two-factor ANOVA whose results showed a significant difference in the *gaze-step* factor [*F*(1, 41) *=* 6.491, *p =* 0.015, *partial η^2^ =* 0.137]. We did not find any significant differences in the *hand-first* factor [*F*(1, 41) *=* 0.039, *p =* 0.843, *partial η^2^ =* 0.001] or in the interaction effects [*F*(1, 41) *=* 1.246, *p =* 0.271, *partial η^2^ =* 0.029].

**Figure 9:**
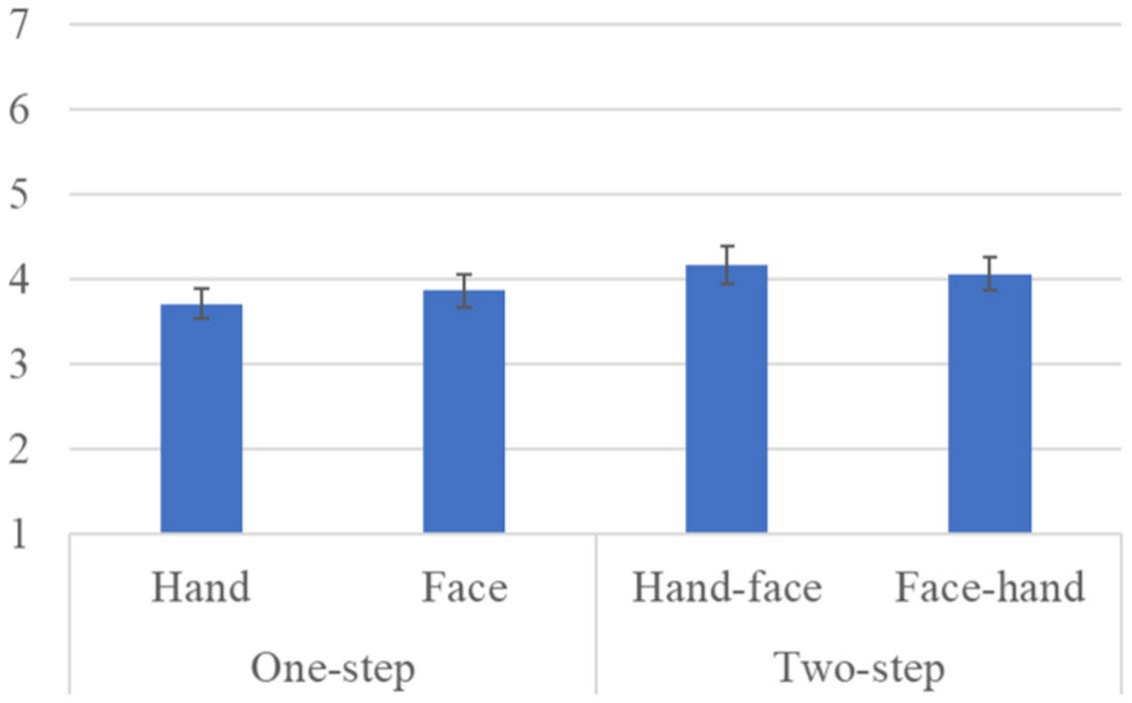
Questionnaire scores and S.E. for likeability.

Figure 9 shows the questionnaire results and the standard error (S.E.) of the likeability scale. We conducted a repeated three-factor ANOVA whose results did not show any significant differences in the *gaze-step* factor [*F*(1, 41) *=* 2.215, *p =* 0.144, *partial η^2^ =* 0.051], in the *hand-first* factor [*F*(1, 41) *=* 0.001, *p =* 1.000, *partial η^2^ =* 0.001], or in the interaction effects [*F*(1, 41) *=* 0.327, *p =* 0.571, *partial η^2^ =* 0.008].

**Figure 10:**
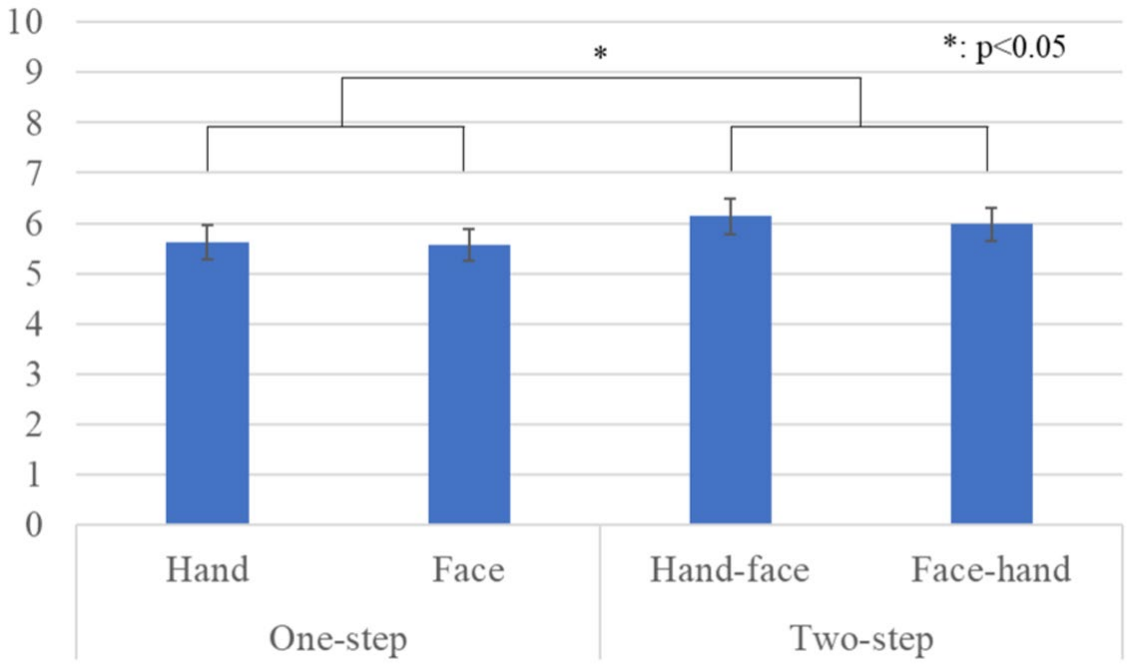
Questionnaire scores and S.E. for naturalness.

Figure 10 shows the questionnaire results and the standard error (S.E.) of the naturalness scores. We conducted a repeated three-factor ANOVA whose results showed a significant difference in the *gaze-step* factor [*F*(1, 41) *=* 5.071, *p =* 0.030, *partial η^2^ =* 0.110]. We did not find any significant differences in the *hand-first* factor [*F*(1, 41) *=* 0.423, *p =* 0.519, *partial η^2^ =* 0.010] or in the interaction effects [*F*(1, 41) *=* 0.164, *p =* 0.687, *partial η^2^ =* 0.004].

### 6.2. Discussion

Eye contact is fundamental for social interaction and has different meanings based on the context and the type of interaction (Emery, 2000). In the cases evaluated outside the FoV, we found a preference for the *two-step* gaze behavior, although we did not find a defined preference in the gaze order. The anthropomorphism and naturalness results are conclusive but not those for likeability. This result still shows the importance of eye contact in interactions with anthropomorphic avatars. Incorporating face-looking behavior into the reaction complements the avatar’s human-looking characteristics (anthropomorphism and naturalness) and satisfies participant expectations. As in human interactions, avoiding direct eye contact might be perceived as a sign that the participant is being ignored or that the avatar does not want to be approached (Argyle and Dean, 1965).

We expected a different behavior when the approach came from outside the FoV because when something enters the cone of vision, the immediate, instinctive reaction is to look at it (Ball et al., 1988; Shahar et al., 2010). Perhaps we obtained different results because the participant’s expectation was based on the avatar’s human-likeness, the expectation of awareness of the avatar, and the importance of face-looking behavior. Experiment 1 argued for the importance of eye contact in the reactions, and the participants probably expected such importance, even though they were approaching from out of sight of the avatar. This result can also be seen in the human-likeness and naturalness differences between *two-step* and *one-step* gaze behaviors. The participants might assume that the avatar is aware of the approach and the intention, both of which might have influenced the participant preferences.

## 7. General discussion

### 7.1. Implications

The possibility of improving how human-looking avatars or robots behave by defining a before-touch reaction seems useful for designing new implementations and improving the present ones. Currently, most studies focus on the effects of touch interactions and after-touch effects. Concerning before-touch behavior, studies are generally more focused on defining when the reaction should take place based on distance, but in this study, we evaluated different reactions based on gaze behavior, FoV, proxemics, and gender. The expected and preferred reactions from a human-looking avatar in a virtual reality environment resemble the expectations of people when interacting with others in the real world. Experiment 1 was defined based on the evaluation of gaze behavior as a reaction in a before-touch situation; Experiment 2 was proposed to evaluate the effect of the FoV in the participant’s perception towards the avatar’s pre-touch reactions. Our results showed that H1 is partially supported. In both experiments, the *two-step* gaze behavior was perceived as more human-like and more natural, and in Experiment 1, it was also more likeable. However, in the likeability item in Experiment 2, the results did not show a particular preference, although in some situations, gaze behaviors were preferred, including face-looking reactions. This result might be due to the differences in the expected behavior and the reaction preferences of each participant. Some might have preferred a more direct and shorter reaction; others would have chosen a more complex and tentative reaction. These findings imply that no matter where the interaction comes from, a human-like avatar reaction must include eye contact.

Based on our results, H2 is also partially supported. Evaluating the influence of FoV in an avatar’s reaction behavior, we found that a *face-first*-looking behavior in the *one-step* condition was more likeable than a *hand-first*-looking behavior for approaches only from inside the FoV, meaning that H2a is partially supported and that H2b is not supported. The results suggest that pre-touch reaction behavior should include a face-looking movement that improves the impression, although the FoV does not strongly define this behavior. Although our results were not conclusive regarding how FoV conditions the expected behaviors, in certain situations, it should be considered, and a face-looking behavior should be prioritized in all cases.

These findings are consistent with the importance of eye contact in human relationships and the likeability of such behavior in approaches from inside FoV. Several studies described the importance of eye contact as an active part of regulating interactions in combination with other nonverbal communication cues (Argyle and Dean, 1965) and getting attention in social communication scenarios (Böckler et al., 2015). Our results support the idea that some nonverbal communication cues in a before-touch situation in human interaction can be applied in scenarios with human-looking agents.

The implementation of face-looking behavior in physical environments is another critical task. In a VR environment, tracking the face position is easy due to the system characteristics used for deployment. But for physical environments, this task can be more complex because an external system might be needed. One option is a motion-capture system, which can provide precise information about the positions of body parts. The such scheme was already used in previous studies involving touch communication situations (Miyashita et al., 2007) and face-looking behavior (Cuello Mejía et al., 2021). Another alternative is using cameras supported by modern computer vision algorithms for the recognition of face and body parts that have been used in several applications, such as the classification of robot interactions based on distances (Feil-Seifer and Matarić, 2011) and catching and juggling with a humanoid robot (Kober et al., 2012).

### 7.2. Contribution and future applications

As said before, most of the current research works related to touch interaction focused on after-touch situations and aim to evaluate the effect after the action has been done, and most of them are also being implemented in the real world, using human-looking robots: robot-initiated touch has a significant effect in people’s responses in a nursing context (Chen et al., 2014), robots that encourage self-disclosure by hug (Shiomi et al., 2017) and using touch interaction information to define the internal state of the robot (Woo et al., 2020). All these studies are done with physical setups and focus on the effect of an already performed touch action.

Similarly, some of those studies search for the combination of real world and virtual reality systems to complement each other, testing situations that could be difficult to evaluate in only one of them. For example, a study aims to develop and enhance Mixed Reality systems to improve communication in Human Robot Interaction (Szafir, 2019) and enhance non-verbal communication in VR environments implementing bidirectional touch (Kotranza et al., 2009). Moreover, a significant trend toward the inclusion of touch stimulation was found regarding the effect on the relaxation feeling and sense of presence in a VR-MIP environment (Serrano et al., 2016). All these show the importance of including non-verbal communication cues when interacting with an agent and how the combination of both scenarios can be extremely beneficial for better communication. Thus, it is important to consider all the possible non-verbal communication information to improve human-agent interaction.

Our study focused on analyzing before-touch situations, and evaluating the factors that could define a pre-touch reaction. Previous studies had checked the effect of personal space in VR environment in relationship with the conveyed emotions (Bönsch et al., 2018) or how touch interaction or facial expressions can persuade the decision-making in an economic bargain (Harjunen et al., 2018), but few studies evaluated the non-verbal communication cues as gaze behavior as pre-touch reactions. Eye contact and avatar’s gaze behavior were found to influence the perception of its human-likeness and likeability. This knowledge can be useful for complementing the current implementations, extending them to the real world with human-looking robots, and improving significantly different kinds of Virtual Reality implementations that need some sort of feedback from the avatar.

### 7.3. Limitations

With our results, we found that our participants preferred an awareness reaction from inside the FoV and relatively complex gaze behavior, although considering other factors that might affect perception would be interesting. For example, using the surrounding space is determined by the cultural background. Some cultures are more likely to approach closer and even engage more easily in touch interactions than others. Some cultures, such as Latin and North Americans, have similarities in their personal space management (Forston and Larson, 1968). But from a broader perspective, such regions as Arabic and Latin American countries are high-contact interaction cultures; Asian and Northern European countries are less open to more intimate social interaction distances (Shuter, 1976; Edinger and Patterson, 1983). Addressing this effect in an avatar’s reaction behavior might be illuminating.

The inclusion of other nonverbal communication cues might also be useful for evaluating the effect on participant’s preferences. For example, by adding appropriate physical touch feedback, immersion can be increased, and a scenario can be perceived as more real. The implemented reactions were only body movements without facial expressions or voice feedback. Adding some surprise effects in the avatar’s behavior depending on the interaction could also be positive. Including such expressions and feedback might spark future studies that verify whether avatars’ interaction feelings and human-like perceptions can be increased.

Moreover, the inclusion of non-verbal communication cues in the interaction with virtual agents could be useful for improving the results of different applications, considering the positive effect they have in other contexts. For example, providing mobile learning platforms based on conversational agents (Troussas et al., 2017, 2019) or systems based on social networks for higher education (Troussas et al., 2020) with an enhanced and interactive interface supported with non-verbal communication cues would be helpful for evaluating the effect of such interaction, leading to a more immersive, efficient and novel learning process.

Although virtual reality is used for studying real world situations, obvious differences exist, which can be challenging for developing systems in one of them whose results are also applicable in the other one. For example, the FoV of the equipment used for deploying VR environments and the distance and spatial perception can differ (Mizuchi and Inamura, 2018; Masnadi et al., 2021). These effects must be considered for developing systems that can be applied in the real world.

## 8. Conclusion

We evaluated the factors that influence a before-touch interaction in a virtual reality environment with a human-like avatar. Experiment 1 implemented four different pre-touch reaction behaviors and evaluated the human-likeness, naturalness, and likeability for each condition by considering gaze-step order, gaze-step number, and avatar gender. Experiment 2 assessed the effect of FoV and evaluated the human-likeness, the naturalness, and the likeability by considering gaze-step number and gaze-step order. A *two-step* gaze reaction behavior was perceived as more human-like and more natural from inside the FoV, and gaze behaviors, which includes a face-looking movement, is preferred over a hand-looking movement. The results from outside the FoV were similar; a two-step gaze reaction behavior was perceived as more human-like and natural. In both experiments, we found no preference regarding the gaze order, but eye contact was essential for improving the interactions. In the context of our proposed interaction, gender did not significantly affect the participants’ perception. In conclusion, we found that gaze behavior as a pre-touch reaction affects the participant’s perception towards the agent, being perceived as more human-like and being more likeable with a two-steps gaze behavior; and that eye contact is fundamental for human-looking agents in a VR environment, regardless the location of the approach. These results will be useful for implementing different behaviors in a virtual reality context and for studying social interactions, increasing the nonverbal information for a more fluent and organic interaction.

## Data availability statement

The raw data supporting the conclusions of this article will be made available by the authors, without undue reservation.

## Ethics statement

The studies involving human participants were reviewed and approved by the ethics committee at the Advanced Telecommunication Research Institute (ATR) (21-501-4). The patients/participants provided their written informed consent to participate in this study.

## Author contributions

DC, HS, and MS: conceptualization, validation, formal analysis and data curation, investigation, resources, writing and original draft preparation, and review and editing. DC and MS: methodology. DC: software and visualization. HS, HI, and MS: supervision. HI and MS: project administration and funding acquisition. All authors have revised, read, and approved the submitted version of the manuscript.

## Funding

This research work was supported by JST CREST Grant, Number JPMJCR18A1, Japan.

## Conflict of interest

The authors declare that the research was conducted in the absence of any commercial or financial relationships that could be construed as a potential conflict of interest.

## Publisher’s note

All claims expressed in this article are solely those of the authors and do not necessarily represent those of their affiliated organizations, or those of the publisher, the editors and the reviewers. Any product that may be evaluated in this article, or claim that may be made by its manufacturer, is not guaranteed or endorsed by the publisher.
