# Supplementary material for: Evaluating gaze behaviors as pre-touch reactions for virtual agents
Source: Front Psychol. 2023 Mar 6;14:1129677. doi: 10.3389/fpsyg.2023.1129677 (PMC10026528; doi:10.3389/fpsyg.2023.1129677)
Supplement: Supplementary file 1 [file Table_1.DOCX]

Supplementary Material

**EVALUATING GAZE BEHAVIORS AS PRE-TOUCH REACTIONS FOR VIRTUAL AGENTS**

Dario Alfonso Cuello Mejía^1,2^*, Hidenobu Sumioka^3^, Hiroshi Ishiguro^2^ and Masahiro Shiomi^1^

^1^ Interaction Science Laboratories, ATR, Kyoto 619-0237, Japan; dario@atr.jp (C.M.), m-shiomi@atr.jp (M.S.)

^2^ Intelligent Robotics Laboratory, Department of Systems Innovation, Graduate School of Engineer Science, Osaka University, Osaka 565-0871, Japan; cuello.dario@irl.sys.es.osaka-u.ac.jp (C.M.), ishiguro@sys.es.osaka-u.ac.jp (H.I.)

^3^ Hiroshi Ishiguro Laboratories, ATR, Kyoto 619-0237, Japan; sumioka@atr.jp (H.S.)

***Correspondence:**Dario Alfonso Cuello Mejía
cuello.dario@irl.sys.es.osaka-u.ac.jp

# Supplementary Data

The questionnaire used for the experiments for evaluating anthropomorphism (Godspeed), likeability (Godspeed) and naturalness (11-point response format item) is the following:

## Japanese version

| 1 | このエージェントの印象は、 | 偽物のような ＿＿＿＿＿＿　自然な |
| --- | --- | --- |
| 2 | このエージェントの印象は、 | 機械的 ＿＿＿＿＿＿ 人間的 |
| 3 | このエージェントの印象は、 | 意識を持たない ＿＿＿＿＿＿ 意識を持つ |
| 4 | このエージェントの印象は、 | 人工的 ＿＿＿＿＿＿ 生物的 |
| 5 | このエージェントの印象は、 | ぎこちない動き ＿＿＿＿＿＿ 洗練された動き |
| 6 | このエージェントの印象は、 | 嫌い ＿＿＿＿＿＿ 好き |
| 7 | このエージェントの印象は、 | 親しみにくい ＿＿＿＿＿＿ 親しみやすい |
| 8 | このエージェントの印象は、 | 不親切な ＿＿＿＿＿＿ 親切な |
| 9 | このエージェントの印象は、 | 不愉快な ＿＿＿＿＿＿ 愉快な |
| 10 | このエージェントの印象は、 | ひどい ＿＿＿＿＿＿ 良い |

あなたに当てはまる第一印象を0から10まで選んでください。

0: 全くそう思わない　5: どちらでもない　10: とてもそう思う

| 11 | このエージェントの触れられる前の振る舞いは、自然だと思う | ＿＿＿＿＿＿＿＿＿＿ |
| --- | --- | --- |

## English version

| 1 | The impression of this agent is、 | Fake ＿＿＿＿＿＿　Natural |
| --- | --- | --- |
| 2 | The impression of this agent is、 | Machine like ＿＿＿＿＿＿ Human like |
| 3 | The impression of this agent is、 | Unconscious ＿＿＿＿＿＿ Conscious |
| 4 | The impression of this agent is、 | Artificial ＿＿＿＿＿＿ Lifelike |
| 5 | The impression of this agent is、 | Moving rigidly ＿＿＿＿＿＿ Moving elegantly |
| 6 | The impression of this agent is、 | Dislike ＿＿＿＿＿＿ Like |
| 7 | The impression of this agent is、 | Unfriendly ＿＿＿＿＿＿ Friendly |
| 8 | The impression of this agent is、 | Unkind ＿＿＿＿＿＿Kind |
| 9 | The impression of this agent is、 | Unpleasant ＿＿＿＿＿＿ Pleasant |
| 10 | The impression of this agent is、 | Awful ＿＿＿＿＿＿ Nice |

Please choose a first impression from 0 to 10 that applies to you.

0: Strongly disagree 5: Neutral 10: Strongly agree

| 11 | The agent’s pre-touch behavior is natural | ＿＿＿＿＿＿＿＿＿＿ |
| --- | --- | --- |
